# The association between immature platelet count and clinical characteristics in pediatric immune thrombocytopenia

**DOI:** 10.1016/j.rpth.2026.103417

**Published:** 2026-03-23

**Authors:** Emily M. Harris, Michele P. Lambert, Amanda Grimes, Jenny Despotovic, Jennifer A. Rothman, Caitlyn McComb, Brian Guedes, Susan Kirk, Abinaya Arulselvan, Matthew Basara, Brenna Cannon, Sam King, Andrew L. Frelinger, Nan Chen, Wendy B. London, Rachael F. Grace

**Affiliations:** 1 Division of Hematology/Oncology, Boston Children's Hospital; Department of Pediatric Oncology, Dana-Farber Cancer Institute; Harvard Medical School, Boston, Massachusetts, USA; 2 Division of Hematology, Children’s Hospital of Philadelphia, Philadelphia, Pennsylvania, USA; 3 Texas Children’s Cancer and Hematology Center; Division of Hematology/Oncology, Department of Pediatrics, Baylor College of Medicine, Houston, Texas, USA; 4 Agios Pharmaceuticals, Cambridge, Massachusetts, USA; 5 Department of Pediatrics, Division of Pediatric Hematology/Oncology, Duke University Medical Center, Duke University, Durham, North Carolina, USA; 6 Division of Pediatric Hematology/Oncology, Dayton Children’s Hospital, Dayton, Ohio, USA; 7 Cancer and Blood Disorders Institute, Johns Hopkins All Children's Hospital, St. Petersburg, Florida, USA; 8 Center for Platelet Research Studies, Dana-Farber/Boston Children's Cancer and Blood Disorders Center, Harvard Medical School, Boston, Massachusetts, USA

**Keywords:** immature platelet count (IPC), immature platelet fraction (IPF), immune thrombocytopenia (ITP), pediatrics, therapeutics

## Abstract

**Background:**

Despite the increasing number of treatments for immune thrombocytopenia (ITP), there are no established laboratory predictors of treatment response. Immature platelet fraction (IPF) and immature platelet count (IPC) are clinical laboratory measurements reflecting bone marrow thrombopoietic activity. The association between IPF and IPC and response to treatments, including thrombopoietin receptor agonists, has not been previously studied in children.

**Objectives:**

This study evaluated the relationship among IPF, IPC, and treatment response in children with ITP.

**Methods:**

This observational cohort study included 4 pediatric medical centers. Inclusion criteria were a diagnosis of ITP, ITP medication treatment, and an available clinical IPF measurement. Laboratory and clinical data were collected via medical record review.

**Results:**

A total of 195 patients were included, with a median age at diagnosis of 7.5 years (range, 0.1-20.7). IPF was inversely associated with platelet count, whereas IPC was positively associated with platelet count. IPC at diagnosis and pretreatment was higher in overall responders than in nonresponders (*P* = .03 and *P* = .001, respectively). There were higher pretreatment platelet counts in overall responders than in nonresponders (*P* < .001), but no differences in IPF at diagnosis or pretreatment between the treatment groups. Pretreatment IPC and platelet count were higher in corticosteroid responders than in nonresponders (*P* = .046 and *P* = .005, respectively), and in intravenous immunoglobulin responders than in nonresponders (*P* = .04 and *P* = .003, respectively). However, pretreatment IPF, IPC, and platelet counts did not differ between thrombopoietin receptor agonist responders and nonresponders.

**Conclusion:**

Higher pretreatment platelet count and IPC correlate with overall treatment response, but the association varies by individual treatment. Biomarkers of treatment response are needed to inform individualized management.

## Essentials

• This study assessed IPF/IPC and treatment outcomes in ITP.
• IPC was higher in corticosteroid and intravenous immunoglobulin responders than in nonresponders.
• IPC was not associated with response to thrombopoietin receptor agonists.
• Future exploration of biomarkers is needed to support individualized treatment strategies for ITP.

## 1 Introduction

Immune thrombocytopenia (ITP) is an acquired disorder characterized by a platelet count < 100 × 10^9^/L due to autoimmune destruction of circulating platelets and impaired thrombopoiesis [1]. The goal of ITP treatment is to increase the platelet count sufficiently to prevent bleeding, decrease fatigue, and/or improve quality of life [2]. Treatments for pediatric ITP include first-line agents such as intravenous immunoglobulin (IVIG) or corticosteroids, and second-line agents such as thrombopoietin receptor agonists (TPO-RAs), intravenous biologic medications such as rituximab, and/or oral immunomodulatory medications [2,3]. In the Pediatric and Adult Registry on Chronic ITP, 38% to 47% of children with ITP who were initially treated with first-line agents required second-line therapies at 6 to 24 months [4]. Although overall response rates are high, patients have variable responses to medications. There are currently no clinical laboratory predictors of treatment response to guide medication selection. Therefore, treatment selection is empiric [2].

Immature platelet fraction (IPF) is a clinically available metric in routine complete blood count testing. Immature platelets containing residual RNA can be stained with a fluorescent dye and measured by flow cytometry, which, when combined with forward light scatter, allows for quantification of immature platelets as a fraction of the total number of platelets in the circulation [5,6]. Immature platelets can also be measured as an absolute immature platelet count (IPC), which is the total platelet number multiplied by IPF [7,8]. IPF and IPC are a function of both bone marrow activity and platelet half-life. TPO-RAs increase platelet production by interacting with the thrombopoietin receptor and stimulating megakaryocytes. We therefore hypothesized that IPF and IPC, which correlate with bone marrow thrombopoietic activity, could serve as surrogate markers of megakaryocyte function and may therefore correlate with response to TPO-RAs.

Thrombocytopenia with an elevated IPF and IPC is most often found in disorders of platelet destruction or consumption. Among patients with ITP, IPF varies but is, on average, higher in patients with ITP than in healthy individuals or in those with disorders such as bone marrow failure [[7], [8], [9], [10]]. Patients with active ITP have a higher IPF than those in remission, which may be due to an increase in platelet half-life as patients achieve remission [11,12]. IPF also positively correlates with the number of lines of treatment used [11,13]. Small retrospective studies of adults with ITP have identified significant differences in pretreatment IPC between patients who responded to corticosteroids and those who did not [14,15]. However, the utility of pretreatment IPF or IPC as potential predictors of treatment response in pediatric patients with ITP has not been established. The objective of this study was to characterize the relationships among pretreatment IPF, IPC, other clinical characteristics, and treatment response in children with ITP.

## 2 Methods

### 2.1 Study population

This observational cohort study was conducted at 4 tertiary pediatric medical centers: Boston Children’s Hospital, Children’s Hospital of Philadelphia, Texas Children’s Hospital, and Duke University Medical Center, and was approved by the Institutional Review Board at each site. Inclusion criteria were a diagnosis of ITP according to the International Classification of Diseases, Ninth Revision (code 287.31) or 10th Revision (code D69.3), availability of a clinical IPF measurement, and treatment with a medication for ITP between January 2010 and December 2020. Patients were excluded if they received more than 1 treatment simultaneously, so that the response to an individual treatment could not be discerned. Patients who received more than 1 treatment sequentially, such that response to each individual treatment could be discerned, were included. For calculating the number of treatments, TPO-RAs were considered a single treatment type, including eltrombopag, romiplostim, and/or avatrombopag.

Demographic, clinical, and laboratory data were collected from a retrospective review of the electronic medical record. For each medication, a “responder” was defined as a patient with a platelet count ≥ 30 × 10^9^/L and at least a 2-fold increase in baseline platelet count within 3 weeks of the first dose of first-line treatments (IVIGs or corticosteroids) or within 3 months of the first dose of second-line treatments (any non-IVIG or corticosteroid treatment) [1]. For patients receiving second-line ITP treatment, platelet laboratory parameters within 1 month of IVIGs or corticosteroid rescue therapy were excluded. Patients who did not meet the response criteria were considered nonresponders. Response was defined for each medication and overall for each patient. Each patient was considered an “overall responder” if they responded to any medication. Treatment dosing and dose-escalation strategies were determined by the treating clinician and varied across patients and centers.

Platelet count, IPF, and IPC at diagnosis were defined as the values measured closest to the time of diagnosis. Pretreatment platelet count, IPF, and IPC were defined as the values measured most IPF immediately before the first dose of the given medication. Platelet laboratory values below the lower limit of detection were treated as missing values.

### 2.2 IPF and IPC measurements

IPF was measured clinically with a Sysmex hematology analyzer that uses fluorescent flow cytometry and a semiconductor diode laser. Platelet RNA was stained with an oxazine fluorescent dye, and the platelets were then passed through a semiconductor diode laser beam. The resulting forward-scatter light and fluorescence intensity were measured as markers of platelet volume and mRNA content, respectively. Immature platelets were distinguished based on fluorescence intensity and light scatter and reported as a fraction of the total platelet count. IPC was calculated by multiplying IPF by the platelet count.

### 2.3 Statistical analysis

A 2-tailed Mann–Whitney U-test was used for comparisons between 2 groups of continuous variables. Chi-square and Fisher’s exact tests were used for comparisons between 2 groups of categorical variables. Simple linear regression was used to evaluate the association between 2 continuous variables. Box-and-whisker plots were generated for platelet count and IPF (at diagnosis and pretreatment) for responders vs nonresponders, overall, and by treatment. A sensitivity analysis excluding patients with secondary ITP was performed to assess the association between pretreatment platelet count and IPC vs response. Depending on the specific study objective, some analyses were performed using medication instances as the experimental unit (multiple medication instances per patient), while others used patients as the experimental unit. *P* values < .05 were considered statistically significant. Statistical analyses were performed and figures generated using Prism v10.0.0 (GraphPad), with confirmation using R version 4.4.1 (R Core Team).

## 3 Results

### 3.1 Demographic and clinical data

IPF and IPC data at diagnosis and/or pretreatment were available for 195 patients treated with ITP-directed medication, with a median age at diagnosis of 7.5 years (range, 0.1-20.7). Demographics and clinical characteristics are summarized in the Table. The median platelet count at diagnosis was 6 × 10^9^/L, with a median IPF at diagnosis of 16.4% and a median IPC at diagnosis of 0.94 × 10^9^/L. Secondary ITP was present in 17.2% (*n* = 33) of patients, including Evans syndrome (*n* = 11), systemic lupus erythematosus (*n* = 3), transplant-associated ITP (*n* = 2), immune hepatitis (*n* = 1), thyroid disease (*n* = 1), common variable immunodeficiencies (*n* = 1), other rheumatologic diagnoses (*n* = 7), and other immunodeficiencies (*n* = 4). Patients with secondary ITP were older at diagnosis than those with primary ITP (median, 13.8 vs 6.4 years; *P* < .001; Supplementary Table S1). Few patients (2.5%, *n* = 4) had a prior splenectomy (Table). There was a median of 1 type of treatment per patient (range, 1-4; Table). The most common medications were IVIGs (62.6%, *n* = 122), corticosteroids (41.0%, *n* = 80), and TPO-RAs (30.8%, *n* = 60). Second-line treatments were used in 51.5% (17/33) of patients with secondary ITP and 34.6% (55/159) of patients with primary ITP.

**Table:** Characteristics of the study cohort (*N* = 195 patients).

Characteristic	*N* = 195 patients	
	*n*	Median (range)
Age at diagnosis (y)	194	7.5 (0.1-20.7)
Platelet count at diagnosis (×10^9^/L)	193	6 (0-141)
IPF (%) at diagnosis	115	16.4 (0.3-47.2)
IPC at diagnosis (×10^9^/L)	115	0.94 (0.008-9.43)
MPV at diagnosis (fL)	48	10.8 (5.4-14.5)

	*n* (%) or *n*
**Sex**	
Male	97 (49.7)
Female	98 (50.3)
**Race**	
Asian	13 (7.3)
Black	12 (6.7)
Caucasian	136 (76.4)
Other	17 (9.6)
Unknown	17
**Ethnicity**	
Hispanic	25 (14.2)
Non-Hispanic	151 (85.8)
Unknown	19
**Secondary ITP**	
Yes	33 (17.2)
No	159 (82.8)
Unknown	3
**Secondary ITP type**	
Evans syndrome	11
SLE	3
Thyroid disease	1
Hepatitis	1
Inflammatory bowel disease	1
Transplant-associated	2
CVID	1
ALPS	1
Other rheumatologic diagnoses^a^	7
Other immunodeficiencies^b^	4
Unspecified	2
**No. of treatments**, median (range)	1 (1-4)
**Medication type**^c^	
IVIG	122 (62.6)
Corticosteroid	80 (41.0)
TPO-RA^d^	60 (30.8)
Rituximab	10 (5.1)
Sirolimus	2 (1)
Mycophenolate	6 (3.1)
6 Mercaptopurine	3 (1.5)
Rho(D) immune globulin	2 (1)
Other^e^	3 (1.5)
Splenectomy	
Yes	4 (2.5)
No	155 (97.5)
Response to any medication	172 (88.2)
Yes	169 (88.0)
No	23 (12.0)
Unknown	3

ALPS, autoimmune lymphoproliferative syndrome; CVID, common variable immune deficiency; IPC, immature platelet count; IPF, immature platelet fraction; ITP, immune thrombocytopenia; IVIG, intravenous immunoglobulin; MPV, mean platelet volume; SLE, systemic lupus erythematosus; TPO-RA, thrombopoietin receptor agonist.

a Other rheumatologic diagnoses include homozygous deletion of CFHR3/CFHR1 (*n* = 1), ANA+ with other rheumatologic symptoms (*n* = 3), mixed connective tissue disorder (*n* = 1), and other connective tissue disorders (*n* = 2).

b Other immunodeficiencies include biallelic ATM mutation (*n* = 1), DiGeorge syndrome 22q11.2 (*n* = 1), chronic immunosuppression after solid organ (bilateral lung) transplant (*n* = 1), and immunosuppressive treatment for Hodgkin’s lymphoma (*n* = 1).

c Many patients received more than 1 medication.

d TPO-RA includes eltrombopag (*n* = 46) and/or romiplostim (*n* = 16) and/or avatrombopag (*n* = 2).

e Other treatments include dapsone (*n* = 1) and hydroxychloroquine (*n* = 2).

There were 169 (88.0%) patients who responded to any medication and 23 (12.0%) who had no response to any medication. Patient response rates were 85.1% (103/121) for IVIGs, 62.3% (48/77) for corticosteroids, and 85% (51/60) for TPO-RAs. Those who were overall responders were statistically significantly younger at diagnosis than those who were overall nonresponders (median, 6.9 years vs 13.0 years; *P* = .003; Supplementary Table S2). There was no difference in the rate of secondary ITP between overall responders and nonresponders (Supplementary Table S2).

### 3.2 Correlation between platelet parameters

**Figure 1:**
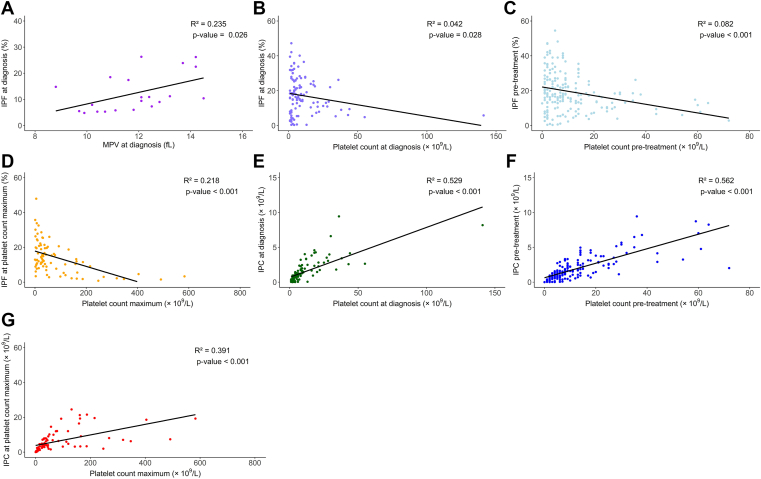
Correlations of laboratory features. (A) Immature platelet fraction (IPF) at diagnosis vs mean platelet volume (MPV) at diagnosis (*n* = 21 patients; *P* = .03). (B) IPF at diagnosis vs platelet count at diagnosis (*n* = 115 patients; *P* = .03). (C) IPF pretreatment vs platelet count pretreatment (*n* = 224 medication instances; *P* < .001). (D) IPF at the time of maximum platelet count vs maximum platelet count (*n* = 96 medication instances; *P* < .001). (E) Immature platelet count (IPC) at diagnosis vs platelet count at diagnosis (*n* = 115 patients; *P* < .001). (F) IPC pretreatment vs platelet count pretreatment (*n* = 224 medication instances; *P* < .001). (G) IPC at the time of maximum platelet count vs maximum platelet count (*n* = 96 medication instances; *P* < .001).

IPF at diagnosis was significantly correlated with mean platelet volume at diagnosis (*P* = .03; Figure 1A). IPF was inversely associated with platelet count at diagnosis (*P* = .03; Figure 1B), pretreatment platelet count, and maximum platelet count (*P* < .001; Figure 1C, D). IPC was positively associated with platelet count at diagnosis, pretreatment platelet count, and maximum platelet count (*P* < .001; Figure 1E–G).

### 3.3 Treatment response and platelet parameters at ITP diagnosis

**Figure 2:**
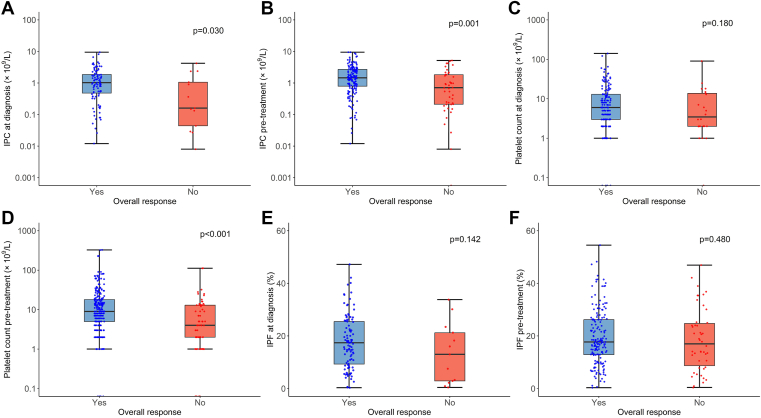
Comparison of platelet metrics between overall treatment responders and nonresponders in the overall cohort. (A) Immature platelet count (IPC) at diagnosis (*n* = 100 responders; *n* = 13 nonresponders; *P* = .03). (B) IPC pretreatment (*n* = 176 responders; *n* = 46 nonresponders; *P* = .001). (C) Platelet count at diagnosis (*n* = 167 responders; *n* = 23 nonresponders; *P* = .18). (D) Platelet count pretreatment (*n* = 264 responders; *n* = 72 nonresponders; *P* < .001). (E) Immature platelet fraction (IPF) at diagnosis (*n* = 100 responders; *n* = 13 nonresponders; *P* = .14). (F) IPF pretreatment (*n* = 176 responders; *n* = 46 nonresponders; *P* = .48).

**Figure 3:**
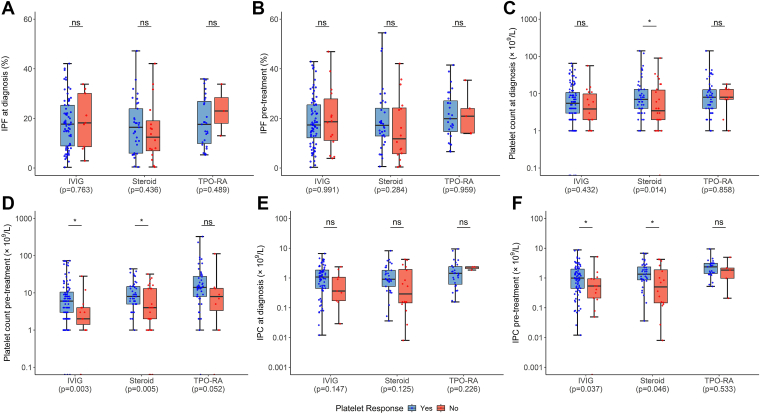
Comparison of platelet metrics between responders and nonresponders to intravenous immunoglobulin (IVIG), corticosteroids (steroid), and thrombopoietin receptor agonists (TPO-RA). (A) Immature platelet fraction (IPF) at diagnosis (IVIG: *n* = 68 vs *n* = 9, *P* = .76; steroid: *n* = 33 vs *n* = 16, *P* = .436; TPO-RA: *n* = 27 vs *n* = 3, *P* = .489). (B) IPF pretreatment (IVIG: *n* = 76 vs *n* = 14, *P* = .99; steroid: *n* = 36 vs *n* = 16, *P* = .28; TPO-RA: *n* = 27 vs *n* = 5, *P* = .96). (C) Platelet count at diagnosis (IVIG: *n* = 102 vs *n* = 18, *P* = .43; steroid: *n* = 48 vs *n* = 28, *P* = .01; TPO-RA: *n* = 50 vs *n* = 9, *P* = .86). (D) Platelet count pretreatment (IVIG: *n* = 102 vs *n* = 17, *P* = .003; steroid: *n* = 46 vs *n* = 28, *P* = .005; TPO-RA: *n* = 49 vs *n* = 9, *P* = .05). (E) Immature platelet count (IPC) at diagnosis (IVIG: *n* = 68 vs *n* = 9, *P* = .15; steroid: *n* = 33 vs *n* = 16, *P* = .13; TPO-RA: *n* = 27 vs *n* = 3, *P* = .23). (F) IPC pretreatment (IVIG: *n* = 76 vs *n* = 14, *P* = .04; steroid: *n* = 36 vs *n* = 16, *P* = .046; TPO-RA: *n* = 27 vs *n* = 5, *P* = .53). ns, not significant.

IPC at diagnosis was significantly higher in overall responders than in overall nonresponders (*n* = 100, median [range], 1.03 [0.01-9.43] × 10^9^/L vs *n* = 13, median [range], 0.16 [0.01-4.21] × 10^9^/L, respectively; *P* = .03; Figure 2A). Platelet count and IPF at diagnosis did not differ significantly between overall responders and nonresponders (Figure 2C, E). Platelet count at diagnosis was significantly higher in patients with secondary ITP (*n* = 33) than in those without secondary ITP (*n* = 157; *P* = .03; Supplementary Table S1). IPF, IPC, and mean platelet volume at diagnosis did not differ significantly between patients with secondary and primary ITP (Supplementary Table S1). Platelet count at diagnosis was significantly higher in corticosteroid responders than in nonresponders (*n* = 48, median [range] platelet count, 7 [1-141] × 10^9^/L vs *n* = 28, median [range] platelet count, 3 (0-90) × 10^9^/L, respectively; *P* = .01), and did not differ significantly between IVIG responders and nonresponders (*n* = 102, median [range] platelet count, 5 [0-65] × 10^9^/L vs *n* = 18, median [range] platelet count, 4 [1-56] × 10^9^/L, respectively; *P* = .43; Figure 3C). IPF and IPC at diagnosis did not differ significantly between responders and nonresponders for any medication (Figure 3A, E).

### 3.4 Treatment response and platelet parameters pretreatment

IPC pretreatment was significantly higher in overall responders than in nonresponders (*n* = 176, median [range] IPC in overall responders, 1.46 [0.01-9.43] × 10^9^/L vs *n* = 46, median [range] IPC in overall nonresponders, 0.71 [0.01-5.15] × 10^9^/L; *P* = .001; Figure 2B), with platelet counts pretreatment also significantly higher in overall responders than in overall nonresponders (*n* = 264, median [range] platelet count in overall responders, 9 [0-325] × 10^9^/L vs *n* = 72, median [range] platelet count in overall nonresponders, 4 [0-112] × 10^9^/L; *P* < .001; Figure 2D). These results were similar when patients with secondary ITP were excluded (Supplementary Figure S1). There was no significant difference in IPF pretreatment between overall responders and nonresponders (Figure 2F).

Patients who responded to corticosteroids had a significantly higher pretreatment IPC than nonresponders (*P* = .046; Figure 3F), a significantly higher pretreatment platelet count (*P* = .005; Figure 3D), and a significantly higher platelet count at diagnosis (*P* = .01; Figure 3C). Patients who responded to IVIGs had a significantly higher pretreatment platelet count than nonresponders (*P* = .003; Figure 3D), and a significantly higher pretreatment IPC than nonresponders (*P* = .04; Figure 3F). Patients who responded to TPO-RAs did not differ significantly in pretreatment platelet count or IPC from nonresponders (Figure 3D, F). IPF pretreatment did not differ significantly between responders and nonresponders for any medication (Figure 3B).

## 4 Discussion

This multicenter study evaluated clinical platelet parameters in patients with ITP prior to treatment with ITP-directed therapies. In this cohort of 195 pediatric patients from 4 tertiary care centers, IPC and platelet count prior to treatment correlated with overall treatment response, particularly with responses to corticosteroids and IVIGs. These parameters at the time of diagnosis were less reliably associated with treatment response, and IPF was not associated with later treatment response at any time point.

When evaluated by treatment type, pretreatment IPC and pretreatment platelet counts were significantly higher in patients who responded to corticosteroids or IVIG than in those who did not. There were no significant differences in IPC between responders and nonresponders to TPO-RAs; however, it is notable that patients treated with TPO-RAs had a high response rate, which limits the analysis based on response. Due to the small numbers of nonresponders, this study may be underpowered to detect modest differences in IPC between TPO-RA responder and nonresponder groups. Additionally, it is possible that patients with chronic or more refractory or symptomatic disease may have been preferentially treated with TPO-RAs. Further study is required in larger cohorts.

The finding that IPC and platelet count are positively associated with response to corticosteroids and IVIGs, but not to TPO-RAs, may be related to differences in the biologic mechanisms of action between these treatment types. IVIGs and corticosteroids cause immune modulation, whereas TPO-RAs act via direct stimulation of thrombopoiesis, which may result in different relationships between IPC and platelet responses. Although IPC and platelet count may be useful for predicting the likelihood of overall treatment response, these data do not support using these parameters or IPF at diagnosis or pretreatment to direct the selection of a particular treatment type, including treatment with TPO-RAs.

Mechanistically, since TPO-RAs stimulate platelet production, it has been hypothesized that these medications may have higher efficacy in individuals with a lower IPF than in those with already significantly increased platelet production. In this cohort, IPF was not predictive of overall treatment response or the likelihood of response to TPO-RAs. However, IPF remains useful as a marker of disease activity and platelet destruction, as evidenced by its inverse relationship with platelet count. The correlation of pretreatment IPC and platelet count with overall treatment response suggests that there may be varying degrees of megakaryocyte dysfunction among responders compared with nonresponders. Higher IPC may be an indicator of a greater likelihood of platelet response to treatment, suggesting it could be prognostically useful in clinical practice. For example, a patient with a high IPC may be more likely to respond to first-line treatment, indicating a greater likelihood of delaying second-line therapies. As IPC incorporates both platelet count and IPF, IPC may be more informative than either platelet count or IPF alone in predicting treatment response in children with ITP who have low peripheral blood platelet counts.

Similar to other studies, these data demonstrate that IPF is consistently higher during periods of lower platelet counts and decreases as the platelet count normalizes. In contrast, platelet count was positively correlated with IPC. IPF and IPC may be informative metrics for tracking disease status over time in children with ITP.

A significant limitation of this study was the small sample size in some treatment groups, which, in part, resulted from IPF data not being uniformly available for all patients at all time points. The availability of IPF/IPC measurements at all included time points may have led to potential selection bias. Analysis was also limited by the high response rate to certain treatments, including TPO-RAs. This study included only patients treated at pediatric centers.

Larger prospective studies are needed to evaluate IPC in combination with other clinical and laboratory parameters in multivariable models to identify the most prognostic biomarkers, which may inform individualized treatment selection for patients with ITP.
