# Supplementary material for: The association between immature platelet count and clinical characteristics in pediatric immune thrombocytopenia
Source: Res Pract Thromb Haemost. 2026 Mar 23;10(3):103417. doi: 10.1016/j.rpth.2026.103417 (PMC13092604; doi:10.1016/j.rpth.2026.103417)

**Supplementary Materials**

**Supplementary Table S1.** Association of laboratory features with secondary ITP (n=192 patients, excluding 3 patients with unknown primary vs secondary ITP status)

|  | N | **Primary ITP***  **(n=159 patients)** | | **Secondary ITP**  **(n=33 patients)** | | p-value |
| --- | --- | --- | --- | --- | --- | --- |
|  |  | n | Median (range) | n | Median (range) |  |
| IPF at diagnosis (%) | 113 | 95 | 16.1 (0.3-42.1) | 18 | 17.5 (2.2-47.2) | 0.775 |
| Platelet count at diagnosis (x 10^9^/L) | 190 | 157 | 5.0 (0-141) | 33 | 10 (1-60) | 0.027 |
| IPC at diagnosis | 113 | 95 | 0.9 (0.01-8.18) | 18 | 1.65 (0.03-9.43) | 0.079 |
| MPV at diagnosis | 47 | 35 | 11.1 (5.4-14.5) | 12 | 10.6 (8.5-14.2) | 0.981 |
| Age at diagnosis, yrs | 191 | 158 | 6.4 (0.1, 19.7) | 33 | 13.8 (0.3, 20.7) | <0.001 |

*Without evidence of secondary cause of ITP at the time of the study

**Supplementary Table S2.** Association of clinical and laboratory features with platelet response to treatment in 195 patients who received 352 medication instances

|  | **Overall platelet response** | | | | |
| --- | --- | --- | --- | --- | --- |
|  | Response | | No response | | p-value |
|  | n (pts) | Median (range) | n (pts) | Median (range) |  |
| Age at diagnosis (years) (n=191) | 168 | 6.9 (0.1-20.7) | 23 | 13.0 (1.3-19.7) | 0.003 |
| Secondary ITP (n=33) | 29 | N/A | 4 | N/A | 1.0 |
| Platelet count at diagnosis  (x10^9^/L) (n=190) | 167 | 6 (0-141) | 23 | 3.0 (0-90) | 0.180 |
| IPF at diagnosis (%) (n=113) | 100 | 17.4 (0.3-47.2) | 13 | 13.0 (0.4-33.8) | 0.142 |
| IPC at diagnosis  (x10^9^/L) (n=113) | 100 | 1.03 (0.01-9.43) | 13 | 0.16 (0.01-4.21) | 0.030 |
| MPV at diagnosis  (fL) (n=47) | 44 | 11.0 (5.4-14.5) | 3 | 10.5 (8.0-13.4) | 0.913 |
|  | **Platelet response to medication** | | | | |
|  | n (med instances)* | Median (range) | n (med instances)* | Median (range) | p-value |
| Platelet count pre-treatment  (x10^9^/L) | 264 | 9 (0-325) | 72 | 4 (0-112) | <0.001 |
| IPF pre-treatment  (%) | 176 | 17.7 (0.3-54.5) | 46 | 17.0 (0.4-46.9) | 0.480 |
| IPC pre-treatment  (x10^9^/L) | 176 | 1.46 (0.01-9.43) | 46 | 0.71 (0.01-5.15) | 0.001 |

^*^ “n (med instances)” refers to number of medication instances rather than number of patients, since patients who received multiple medications at different times have multiple pre-treatment values.

**Supplementary Figure S1**. Comparison of platelet metrics between overall treatment responders and non-responders excluding the patients with secondary ITP **A**. IPC pre-treatment (n=142 responders; n=37 non-responders, p=0.037) **B**. Platelet count pre-treatment (n=215 responders; n=55 non-responders, p=0.002). These are analogous to Figures 2B and 2D in the overall cohort.


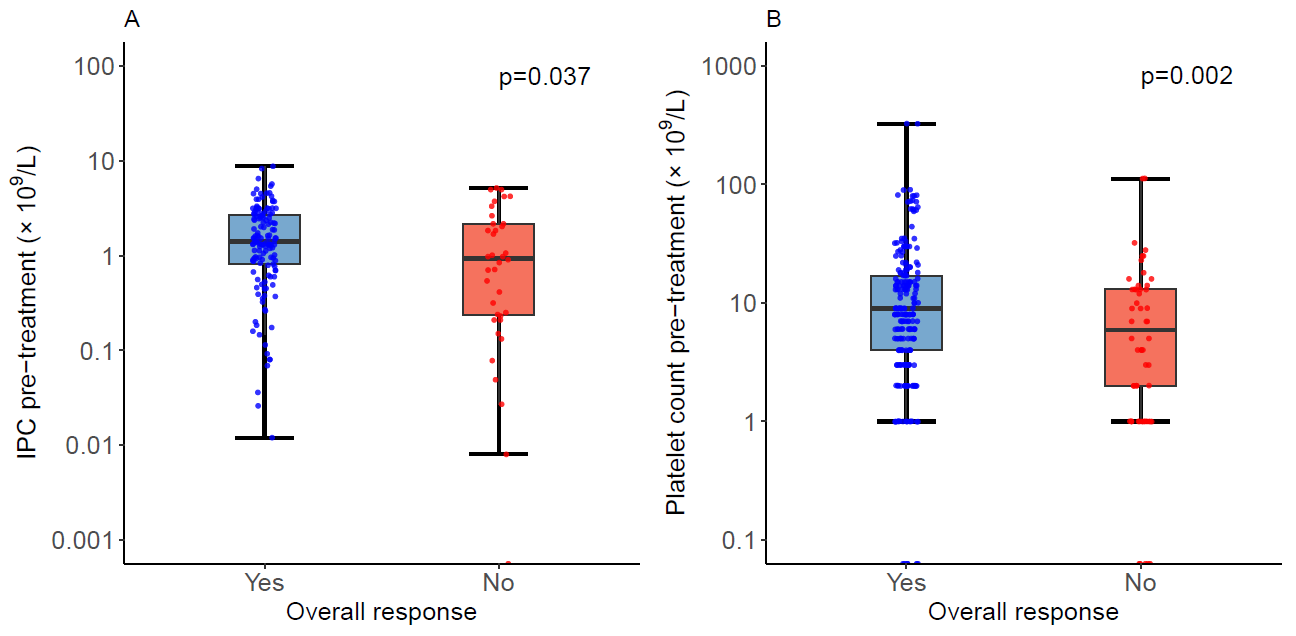

Supplement: Supplementary Material [file mmc1.docx]
